# 

**Published:** 1898-10

**Authors:** 


### BOOKS RECEIVED.

A Manual of Otology. By Gorham Bacon, A. M., M. D., Professor of Otology in Cornell University Medical College, New York. With an introductory chapter by Clarence J. Blake, M. D., Professor of Otology in the Harvard Medical School, Boston. In one handsome 12mo volume of 400 pages, with 109 engravings and 1 colored plate. Cloth, $2 net. Philadelphia and New York: Lea Brothers & Co., Publishers.

The Essentials of Histology. By Edward A. Schafer, F. R. S., Professor of Physiology in University College, London. New (5th) edition. Revised and enlarged. Octavo, 350 pages, with 325 illustrations. Cloth, $3 net. Philadelphia and New York: Lea Brothers & Co., Publishers.

A Text-Book of Practical Therapeutics: With Especial Reference to the Application of Remedial Measures to Disease and their Employment upon a Rational Basis. By Hobart Amory Hare, M. D., Professor of Therapeutics and Materia Medica in the Jefferson Medical College of Philadelphia. With special chapters by Drs. G. E. deSchweinitz, Edward Martin and Barton C. Hirst. New (7th) edition. In one 8vo volume of 770 pages, illustrated. Cloth, $3.75; leather, $4.50 net.

Transactions of the State Medical Society of Wisconsin for the Year 1898. Edited by Charles S. Sheldon, M. D., Secretary. Octavo, pp. xiv.—583. Madison, Wis.: Tracy, Gibbs & Co., Printers. 1898.

Elements of Histology. By E. Klein, M. D., F. R. S., Lecturer on General Anatomy and Physiology in the Medical School of St. Bartholomew's Hospital, London, and J. S. Edkins, M. A., M. B., Joint Lecturer on and Demonstrator of Physiology in the Medical School of St. Bartholomew's Hospital, London. In one 12mo volume of 506 pages, with 296 illustrations. Cloth, $2 net. Philadelphia and New York: Lea Brothers & Co., Publishers.

A Clinical Text-Book of Medical Diagnosis. Based on the Most Recent Methods of Examination. By Oswald Vierordt, M. D., Professor of Medicine at the University of Heidelberg. Translated, with the author's permission, from the fifth enlarged German edition by Francis H. Stuart, A. M., M. D. Fourth American edition, from the fifth German. Handsome royal octavo volume of over 600 pages, with 194 illustrations, many of them in colors. Prices, cloth, $4.00 net; sheep or half-morocco, $5.00 net. Philadelphia: W. B. Saunders, 1898.

A Text-Book of Materia Medica, Therapeutics and Pharmacology. By George F. Butler, Ph. G., M. D., Professor of Materia Medica and of Clinical Medicine in the College of Physicians and Surgeons, Chicago. Second edition, thoroughly revised. Handsome octavo volume of 860 pages, illustrated. Prices, cloth, $4.00 net; sheep, $5.00 net. Philadelphia: W. B. Saunders. 1898.

An American Text-Book of Gynecology, Medical and Surgical. Edited by J. M. Baldy, M. D., Professor of Gynecology in the Philadelphia Polyclinic, etc. Second edition, thoroughly revised. Handsome imperial octavo volume of 718 pages, with 341 illustrations in the text and thirty-eight colored and half-tone plates. Prices, cloth, $6.00 net; sheep or half-morocco, $7.00 net. Philadelphia: W. B. Saunders, 1898.

An American Text-Book of the Diseases of Children. Edited by Louis Starr, M. D., Consulting Pediatrist to the Maternity Hospital, Philadelphia. Second edition, revised and enlarged. Handsome imperial octavo volume of 1,244 pages, profusely illustrated. Prices, cloth, $7.00 net; sheep or half-morocco, $8.00 net. Philadelphia: W. B. Saunders. 1898.

Pathology and Morbid Anatomy. By T. Henry Green, M. D., Lecturer on Pathology and Morbid Anatomy at Charing-Cross Hospital Medical School, London. New (eighth) American from the eighth and revised English edition. In one very handsome royal octavo volume of 600 pages, with 215 engravings, many being new, and a colored plate. Cloth, $2.50 net. Philadelphia and New York: Lea Brothers & Co., Publishers.

Diseases of Women: A Manual of Gynecology. Designed especially for the use of students and general practitioners. By Francis H. Davenport, M. D., Assistant Professor of Gynecology in the Medical Department of Harvard University, Boston. New (third) revised and enlarged edition. In one handsome 12mo volume of 387 pages, with 155 illustrations. Cloth, $1.75 net. Philadelphia and New York: Lea Brothers & Co.

Transactions of the Medical Society of the State of New York. For the year 1898. Edited by Frederic C. Curtis, M. D., Secretary, Albany, N. Y. Octavo, pp. viii.—512. Philadelphia: Wm. J. Dornan, Printer. 1898.

Transactions of the Ohio State Medical Society at its Forty-third Annual Meeting, held at Columbus, May 4, 5 and 6, 1898. Edited by Dr. P. Maxwell Foshay. Small 8vo, pp. 558. Cleveland, O.: J. B. Savage Press. 1898.

Transactions of the American Surgical Association. Volume XVI. Edited by De Forest Willard, A. M., M. D., Ph. D., Recorder for the Association. Octavo, pp. xlii.—318. Philadelphia: William J. Dornan, Printer. 1898.

Manual of Chemistry. A guide to lectures and laboratory work for beginners in chemistry. A text-book specially adapted for students of pharmacy and medicine. By W. Simon, Ph. D., M. D., Professor of Chemistry and Toxicology, College of Physicians and Surgeons, Baltimore; Professor of Chemistry in the Maryland College of Pharmacy. New (sixth) edition. In one 8vo volume of 532 pages, with forty-six engravings and eight colored plates, illustrating sixty-four of the most important chemical tests. Price, cloth, $3.00 net. Philadelphia and New York: Lea Brothers & Co., Publishers.

Manual of Skin Diseases. With special reference to diagnosis and treatment. For the use of students and general practitioners. By W. A. Hardaway, M. D., Professor of Diseases of the Skin in the Missouri Medical College, St. Louis. Second edition, entirely rewritten and much enlarged. In one handsome 12mo volume of 560 pages, with forty engravings and two colored plates. Cloth, $2.25 net. Philadelphia and New York: Lea Brothers & Co., Publishers.

A Treatise on the Science and Practice of Midwifery. By W. S. Playfair, M. D., LL. D., F. R. C P., Emeritus Professor of Obstetric Medicine in King's College, London; Examiner in Midwifery to the Universities of Cambridge and London. Seventh American from the ninth English edition. In one very handsome 8vo volume of 700 pages, with 207 engravings and seven full-page plates. Cloth, $3.75 net; leather, $4.75 net. Philadelphia and New York: Lea Brothers & Co., Publishers.

Medical Diseases of Infancy and Childhood. By Dawson Williams, M. D., Physician to the East London Hospital for Children. In one 12mo volume of 629 pages with eighteen illustrations. Cloth, $2.50 net. Philadelphia and New York: Lea Brothers & Co., Publishers.

A Guide to the Clinical Examination and Treatment of Sick Children. By John Thomson, M. D., Extra Physician to the Royal Hospital for Sick Children, London; Lecturer on Diseases of Children, Edinburgh School of Medicine. In one crown 8vo volume of 350 pages, with fifty-two illustrations. Cloth, $1.75 net. Philadelphia and New York: Lea Brothers & Co., Publishers.

